# The ability of physical activity in reducing mortality risks and cardiovascular loading and in extending life expectancy in patients with COPD

**DOI:** 10.1038/s41598-021-00728-2

**Published:** 2021-11-04

**Authors:** Chin-Chung Shu, June-Han Lee, Min-Kuang Tsai, Ta-Chen Su, Chi Pang Wen

**Affiliations:** 1 Department of Internal Medicine, National Taiwan University Hospital, Taipei, Taiwan; 2 Institute of Population Health Sciences, National Health Research Institutes, Zhunan, Taiwan; 3 Environmental and Occupational Medicine, National Taiwan University Hospital, Taipei, Taiwan

**Keywords:** Chronic obstructive pulmonary disease, Health policy

## Abstract

For chronic obstructive pulmonary disease (COPD), the role of physical activity in reducing COPD mortality and heart loading and in extending life expectancy remains unclear. Participants in comprehensive medical screening were recruited with spirometry on everyone. We analyzed physical activity volume calculated from intensity, duration and frequency of self-reported exercise history. Deaths were identified from the National Death File. The impacts of physical activity on mortality, heart rate and life expectancy were analyzed. Among the cohort of 483,603 adults, 32,535 had spirometry-determined COPD, indicating an adjusted national prevalence of 11.4% (male) and 9.8% (female). On the average, COPD increased all-cause mortality with a hazard ratio of 1.44 and loss of 6.0 years in life expectancy. Almost two thirds (65%) of the causes of deaths were extra-pulmonary, such as cardiovascular disease, diabetes, cancer and kidney diseases. In addition, COPD was associated with increases in heart rate proportionate to its severity, which led to higher mortality. Participants with COPD who were fully active physically could reduce mortality and have improved heart rates as compared with those without physical activity. In addition, their life expectancy could be extended close to those of the no COPD but inactive cohort. Fully active physical activity can help patients with COPD overcome most of the mortality risks, decrease heart rate, and achieve a life expectancy close to that of patients without COPD. The effectiveness of physical activity on COPD is facilitated by its systemic nature beyond lung disease.

## Introduction

Chronic obstructive pulmonary disease (COPD) remains a common disease and accounts for a global disease burden of more than 300 million worldwide^1,2^. Notably, COPD-related mortality is still increasing despite improvements in medicine, and it is close to becoming the third leading cause of death in the world^1,3^. The case fatality rate of patients with COPD is high, with 7% and 14% reported within a 2- and 3-year span, respectively^4,5^. The causes of death are not only directly from COPD but also from outside the lungs, especially cardiovascular disease (CVD)^6^. In fact, CVD is one of the most important extrapulmonary comorbidities^7^. The attributable proportion of deaths from CVD could be as high as 16–39%^5,8^.

Treating COPD with medication has not been satisfactory, as the Global Initiative for Chronic Obstructive Lung Disease (GOLD) stated that “there is a lack of high quality evidence to support initial pharmacological treatment” (2020 GOLD)^9^. In non-pharmacologic therapy, smoking cessation has been the mainstay for smokers with COPD. Other effective strategies to reduce mortality or to improve the outcome of COPD are rarely reported, and regular leisure time physical activity (exercise) as a non-pharmacological treatment emerged as an essential management to explore because patients with COPD have low levels of physical activity^10,11^, which is correlated with mortality^12^.

Notably, COPD-specific physical activity has been limited to pulmonary rehabilitation because we still consider COPD a “lung” disease. Studies to reduce all-cause mortality by physical activity remained limited, and most recruited small samples^13–16^. While the fact that life expectancy is shortened by COPD is universally understood and mortality risks are taken for granted, few researchers, including the GOLD initiative and Cochrane review^9,11^, have indicated that it could be reversed by physical activity. With the availability of a large cohort of nearly half a million subjects from a health surveillance program and with exercise volume calculated for each individual^17^, we studied the systemic effect of leisure time physical activity on patients with COPD.

## Methods

### Participant enrollment

In this historically prospective cohort study, all subjects aged 20 years or older were recruited when they participated in a standard health-screening program run by a private firm (MJ Health Management Institution, Taipei, Taiwan) between 1994 and 2008. The study was approved by the Institutional Review Boards at the National Health Research Institutes, Taiwan (IRB No: EC0981201-E), and all methods were performed in accordance with relevant guidelines and regulations. Informed consent was obtained from all participants to authorize the processing and analysis of the data. A study cohort of 483,603 participants with blood tests and lung function data was enrolled. Participants had received screening spirometry (HI-501, HI-701, or HI-801; Chest M.I. Inc., Tokyo, Japan). In addition, participants completed a comprehensive health history questionnaire to collect information on smoking, education and physical activity since 1996. The details of the MJ cohort were reported previously^18^, and around half of the participants returned for second health examination and questionnaire.

### Data collection

COPD was defined as observed forced expiratory volume in one second (FEV1)/forced vital capacity (FVC) < 0.70 in screening spirometry. The status of COPD was categorized into four stages, which were modified according to criteria from the GOLD^19^. Stage I denoted observed FEV_1_ ≥ 80% of predicted value; Stage II was 50% ≤ observed FEV1 < 80%; Stage III was 30% ≤ observed FEV1 < 50%; and Stage IV represented observed FEV_1_ < 30%. We then calculated the national COPD prevalence by adjusting the prevalence of this cohort using gender and age (the weight of the average age in a five-year range according to the 2017 Report of the Ministry of the Interior, Taiwan).

On the questionnaire, smoking status was defined according to the answer to "Do you currently smoke cigarettes?" Leisure time regular exercise volume was ascertained by three multiple choice questions. The intensity of weekly regular exercise was converted into MET (Metabolic Equivalent for Task) per week^17^. The amount of weekly exercise was intensity (METs) multiplied by the duration (hours) per week and classified into three levels: “inactive”, for exercise of < 15 min a day, or < 3.75 MET-hours/week; “low active”, for exercise of 15–29 min a day on average; or 3.75–7.49 MET-hours/week; and “fully active”, for exercise ≥ 30 min a day or ≥ 7.5 MET-hours/week, which fully met the current exercise recommendation of ≥ 150 min/week^20^.

### Follow-up

We obtained follow-up data for all participants in the study from the National Death File registry between 1997 and 2008 by cross-referencing their national identification numbers. A total of 8,458 deaths were identified with a median follow-up period of 8.8 years. Causes of death were classified according to International Classification of Diseases, 9th version (ICD-9).

### Statistical analysis

Cox proportional hazards regression analysis was used to identify significant predictors in multivariate models. Hazard ratios (HRs) and 95% confidence intervals (CIs) were estimated for each variable. HRs were calculated using the Cox proportional hazards model, with adjustments made as appropriate for confounders. Life expectancy was calculated as previously described^21,22^. All statistical tests were two-sided with the alpha level set at 0.05 and all statistical analyses were performed in SAS 9.4 (SAS Institute Inc., Cary, NC).

## Results

### Participants’ clinical characteristics

During the study period, 483,603 participants were recruited and analyzed (Table 1 and Fig. A1 in Appendix file). The participants were predominantly aged 20–39 years (51.7%) and then 40–64 years (40.2%). Males comprised 47.2%, and the non-smoking proportion was 70.3%. Over half (53.5%) were inactive, about one fifth (22.0%) were low active, and about one quarter (24.5%) were fully active, and the rates were similar in the COPD and non-COPD groups. Hypertension and diabetes mellitus, risk factors for CVD, accounted for 18.6% and 5.5%, respectively, and were higher in participants with COPD than in those without. In addition, we analyzed the heart rate for CVD loading and found that the resting heart rates and the tachycardia percentage (≥ 100/min) were higher (2.6% vs. 1.5%, *p* < 0.05) in participants with COPD than in those without COPD (Table 2).

**Table 1:** Characteristics of the cohort by COPD status.

	Total		Non-COPD		COPD		COPD stages							
	No	%	No	%	No	%	Stage 1		Stage 2		Stage 3		Stage 4	
							No	%	No	%	No	%	No	%
Total	483,603	(100.0)	371,493	(71.2)	32,535	(8.5*)	4999	(1.7)	18,500	(4.5)	7218	(1.8)	1818	(0.5)
**Gender**														
Male	228,178	(100)	178,245	(73.0)	16,582	(8.9*)	2790	(1.8)	9342	(4.8)	3531	(1.8)	919	(0.5)
Female	255,426	(100)	193,249	(69.5)	15,953	(8.0*)	2209	(1.7)	9158	(4.5)	3687	(1.8)	899	(0.5)
**Age**														
20–39	250,134	(51.7)	216,274	(58.2)	11,872	(36.5)	950	(19.0)	7185	(38.8)	3124	(43.3)	613	(33.7)
40–64	194,221	(40.2)	135,515	(36.5)	14,042	(43.2)	2143	(42.9)	8313	(44.9)	2817	(39.0)	769	(42.3)
≧65	39,248	(8.1)	19,704	(5.3)	6621	(20.3)	1906	(38.1)	3002	(16.2)	1277	(17.7)	436	(24.0)
**Education**														
≦Middle school	113,122	(27.9)	70,297	(21.7)	8707	(48.7)	1698	(53.7)	4596	(45.8)	1798	(49.1)	615	(60.2)
≧High school	292,044	(72.1)	253,608	(78.38)	9177	(51.3)	1466	(46.3)	5441	(54.2)	1863	(50.9)	407	(39.8)
**BMI**														
< 18.5	37,920	(7.8)	29,789	(8.0)	2165	(6.6)	227	(4.5)	1159	(6.3)	615	(8.5)	164	(9.0)
18.5–24.9	310,487	(64.2)	246,110	(66.2)	20,714	(63.7)	3273	(65.5)	11,837	(64.0)	4499	(62.3)	1105	(60.8)
25.0–29.9	115,866	(24.0)	82,700	(22.3)	8305	(25.5)	1313	(26.3)	4759	(25.7)	1770	(24.5)	463	(25.5)
≧30	19,331	(4.0)	12,895	(3.5)	1351	(4.2)	186	(3.7)	745	(4.0)	334	(4.6)	86	(4.7)
**Smoking status**														
Never	274,182	(70.3)	220,812	(70.4)	10,112	(62.2)	1810	(61.6)	5656	(62.1)	2047	(62.1)	599	(64.5)
Ex-smoker	24,582	(6.3)	19,187	(6.1)	1502	(9.2)	324	(11.0)	796	(8.7)	288	(8.7)	94	(10.1)
Current smoker	91,505	(23.4)	73,742	(23.5)	4654	(28.6)	803	(27.3)	2652	(29.1)	963	(29.2)	236	(25.4)
**Drinking status**														
Never or occasional	298,607	(77.2)	242,059	(77.8)	11,216	(68.2)	2007	(68.2)	6275	(68.1)	2278	(67.8)	656	(70.8)
Regular drinker	88,175	(22.8)	68,891	(22.2)	5220	(31.8)	936	(31.8)	2933	(31.9)	1081	(32.2)	270	(29.2)
**Leisure time exercise**														
Inactive	220,814	(53.5)	175,246	(53.2)	9203	(50.4)	1433	(44.3)	5184	(50.6)	2002	(53.4)	584	(55.3)
Low active	91,016	(22.0)	74,665	(22.7)	4068	(22.3)	650	(20.1)	2373	(23.2)	830	(22.2)	215	(20.4)
Fully active	101,019	(24.5)	79,279	(24.1)	5003	(27.4)	1152	(35.6)	2680	(26.2)	914	(24.4)	257	(24.3)
**Hypertension**														
No	393,412	(81.4)	316,582	(85.2)	23,650	(72.7)	3213	(64.3)	13,930	(75.3)	5323	(73.7)	1184	(65.1)
Yes	90,185	(18.6)	54,907	(14.8)	8885	(27.3)	1786	(35.7)	4570	(24.7)	1895	(26.3)	634	(34.9)
**Diabetes mellitus**														
No	456,952	(94.5)	356,036	(95.8)	30,059	(92.4)	4532	(90.7)	17,137	(92.6)	6742	(93.4)	1648	(90.6)
Yes	26,633	(5.5)	15,445	(4.2)	2474	(7.6)	466	(9.3)	1363	(7.4)	475	(6.6)	170	(9.4)

All percentages are calculated within the same subgroup by different characteristics except the first row, which is percentage of subgroup by total population.

*Gender- and age-adjusted national prevalence. Age adjusted means that the prevalence of the present cohort has been adjusted by the weight of average age in a five-year range according to 2017 Report of Ministry of the Interior, Taiwan.

**Table 2:** Distribution of heart rate by COPD stages.

	Total		All-cause mortality	Non-COPD		COPD		COPD stages							
	No	%	HR (95% CI)	No	%	No	%	Stage 1		Stage 2		Stage 3		Stage 4	
								No	%	No	%	No	%	No	%
Heart rate 40–59 /min	36,818	(7.6)	0.98 (0.91–1.07)	29,188	(7.9)	2223	(6.8)	429	(8.6)	1268	(6.9)	426	(5.9)	100	(5.5)
60–69	139,973	(29.0)	1.00	110,738	(29.8)	8052	(24.8)	1389	(27.8)	4644	(25.1)	1649	(22.9)	370	(20.4)
70–79	179,945	(37.2)	1.08 (1.03–1.13)	139,263	(37.5)	11,937	(36.7)	1781	(35.6)	6907	(37.4)	2634	(36.5)	615	(33.9)
80–89	91,245	(18.9)	1.25 (1.18–1.32)	67,553	(18.2)	7272	(22.4)	1016	(20.3)	4113	(22.2)	1687	(23.4)	456	(25.1)
90–99	26,342	(5.5)	1.64 (1.52–1.77)	18,537	(5.0)	2171	(6.7)	275	(5.5)	1151	(6.2)	578	(8.0)	167	(9.2)
100–109	6841	(1.4)	2.23 (2.01–2.48)	4573	(1.2)	631	(1.9)	71	(1.4)	310	(1.7)	180	(2.5)	70	(3.9)
110–139	2021	(0.4)	2.23 (1.86–2.67)	1288	(0.3)	229	(0.7)	35	(0.7)	94	(0.5)	62	(0.9)	38	(2.1)
80–99 (High normal)	117,587	(24.3)	1.34 (1.27–1.41)	86,090	(23.2)	9443	(29.0)	1291	(25.8)	5264	(28.5)	2265	(31.4)	623	(34.3)

Hazard ratios were adjusted for age, gender, education, BMI, smoking status, physical activity, anemia, blood pressure and blood glucose.

In all, 371,493 (76.8%) participants had normal pulmonary function, and 32,535 (6.7%) had COPD. Of these, 10,112 (62.2%) were non-smokers, while 6156 (37.8%) were ever smokers. After standardization by national average age, the estimated national incidences of COPD were 8.9% in men and 8.5% in women (Fig. A2 in Appendix file). The adjusted estimated prevalences were 11.4% (male) and 9.8% (female) after adjustment for those age 40 or older. Among participants with COPD, stage 1 was identified in 1810 (15.4% of COPD), stage 2 in 5656 (56.9%), stage 3 in 2047 (22.2%), and stage 4 in 599 (5.6%) (Table 1).

### Risk of mortality by COPD

#### For different causes of death

In a median of 8.8-years of follow up, there were 11,989 deaths due to any cause. Of these, those without COPD had 8458 (2.28%) deaths, and participants with COPD had 3531 deaths (10.85%). Cox proportional hazard regression showed that the HRs of all-cause mortality significantly increased in all participants with COPD (HR: 1.44) compared with those without COPD after adjustment for age, gender, education, BMI, anemia, hypertension and blood glucose (Table 3). All causes of death were higher in participants with COPD than in those without COPD, including cancer (HR: 1.11), CVD (HR: 1.55), infectious disease (HR: 1.97), respiratory disease (HR: 3.22), disease of digestive (HR: 1.45) and genitourinary system (HR: 1.57), diabetes mellitus (HR: 1.45), and expanded CVD (HR 1.55). With regard to the categories of the causes of death, 65% were extra-pulmonary causes (Table A1 in Appendix).

**Table 3:** Mortality risk for different chronic obstructive pulmonary disease stages by cause of death.

Cause of death	Non-COPD		COPD			COPD											
						Stage 1			Stage 2			Stage 3			Stage 4		
	Death	HR	Death	HR		Death	HR		Death	HR		Death	HR		Death	HR	
All causes	8458	1.00	3531	1.44	*	691	1.15	*	1678	1.40	*	831	1.69	*	331	2.44	*
Male	5134	1.00	2359	1.46	*	448	1.12		1124	1.45	*	561	1.75	*	226	2.65	*
Female	3324	1.00	1172	1.38	*	243	1.22		554	1.30	*	270	1.57	*	105	2.08	*
All cancer	3666	1.00	1134	1.11	*	239	0.94		585	1.24	*	249	1.23	*	61	1.15	
Lung cancer	688	1.00	301	1.42	*	60	1.25		148	1.25		75	1.64	*	18	1.52	
Non lung cancer	2978	1.00	833	1.03	*	179	0.84		437	1.17	*	174	1.13		43	1.05	
Cardiovascular disease	1495	1.00	781	1.55	*	158	1.23		365	1.48	*	178	1.75	*	80	3.38	*
Ischemic heart disease	421	1.00	208	1.58	*	39	1.06		91	1.48	*	52	1.82	*	26	3.88	*
Stroke	620	1.00	332	1.55	*	67	1.44	*	165	1.57	*	70	1.53	*	30	3.01	*
Infectious disease	100	1.00	72	1.97	*	17	1.62		23	0.96		25	4.49	*	7	5.24	*
Respiratory system	326	1.00	424	3.22	*	53	1.61	*	151	2.32	*	137	5.39	*	83	10.47	*
COPD	68	1.00	242	11.78	*	21	4.37	*	66	5.95	*	92	20.69	*	63	48.44	*
Pneumonia	165	1.00	119	1.74	*	22	1.21		55	1.82	*	26	1.86	*	16	4.59	*
Digestive system	578	1.00	221	1.45	*	43	1.16		116	1.61	*	35	1.16		27	3.26	*
Genitourinary system	229	1.00	116	1.57	*	25	1.41		51	1.17		28	2.40	*	12	4.54	*
Kidney diseases	185	1.00	99	1.82	*	22	1.71		42	1.36		25	2.42		10	5.32	*
Diabetes mellitus	461	1.00	259	1.45	*	52	2.71	*	125	2.59	*	60	4.20	*	22	0.97	
Expanded CVD	2141	1.00	1139	1.55	*	232	1.26	*	532	1.49	*	263	1.85	*	112	2.91	*

HRs are compared with the reference group of all Non-COPD. *CVD* cardiovascular disease. Expanded CVD: CVD plus diabetes plus kidney disease. HRs were adjusted for age, gender, education, BMI, anemia, hypertension and blood glucose. *p < 0.05.

#### For different stages of COPD

Regarding the association between death and severity of COPD (Table 3), the HRs for all-cause mortality increased with COPD stage, from stage 1 (HR: 1.15) to stage 2 (HR: 1.40), stage 3 (1.69) and stage 4 (HR: 2.44). Even in stage 1 COPD, significantly higher HRs could be found in all-cause mortality (HR 1.15), and causes of death included expanded CVD, stroke, diabetes mellitus, respiratory disease and COPD.

#### Validation by smoking, gender and participants with second health examinations

We stratified the mortality risk by sex and smoking (Tables A2 and A3 in Appendix). The adjusted HRs for all-cause mortality were 1.46 for the male and 1.38 for the female subgroups (both *p* < 0.05) (Table A2 in Appendix). The trends were similar in both the smoking and non-smoking subgroups (Tables A2 and A3 in Appendix). For participants with a second round of health examination (n = 206,117), the mortality risks were similar for different COPD stages and genders (Table A4 in Appendix).

### Impact of regular physical activity on mortality and life expectancy in subjects with COPD

**Figure 1:**
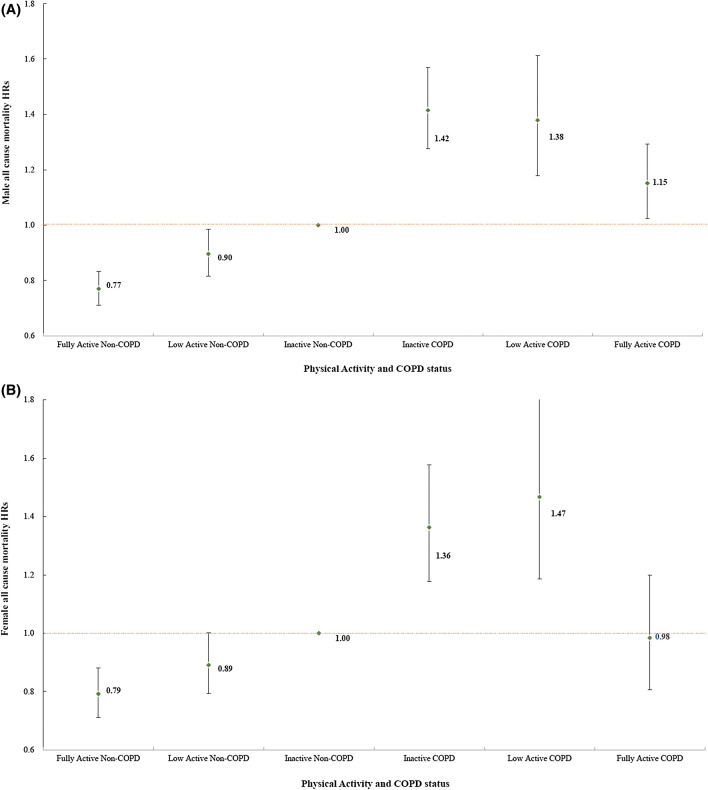
Male (**A**) and Female (**B**) hazard ratios (HR) for all-cause mortality risk by chronic obstructive pulmonary disease (COPD) and Leisure time exercise status. HRs were adjusted for age, gender, education, body mass index, smoking habit, anemia status, hypertension, and fasting blood glucose.

After adjustment for age, gender, education, BMI, and underlying diseases, it was found that fully active physical activity could reduce all-cause mortality in participants with COPD as compared with those who were inactive and had COPD (HR: from 1.40 to 1.10 [*p* < 0.05 between the two subgroups]) (Table 4, and Fig. 1). The all-cause mortality in the fully active COPD group was similar to that of the inactive no-COPD group (Fig. 1 and Table A5 in the Appendix). In addition, HRs of death due to all causes, CVD, cancer, and expanded CVD could be reduced by fully active exercise in the COPD group as compared with the inactive COPD group (Table 4 and Table A5 in Appendix). Only death by respiratory disease was still higher in the COPD group than in the inactive COPD group (HR: 2.13 [1.5–3.0]) (Table 4). The effects of mortality reduction in CVD, expanded CVD (kidney disease and diabetes) and all-cause mortality were similar in the non-COPD group but could be reduced by low active exercise in the non-COPD group (Table 4). This finding indicated that exercise predominantly reduced extrapulmonary causes of death in patients with COPD. Those with COPD who were physically active also had lower heart rates than those of patients with COPD who were inactive (Table A6 in Appendix) and had high normal heart rates (80–99/min) or resting tachycardia (≥ 100/min), which led to higher mortality than that of participants with resting heart rates of 60–69/min (Table A7 in Appendix).

**Table 4:** The comparison of mortality risk by amount of leisure time exercise between participants with or without COPD.

Cause of death	Leisure time exercise														
	Inactive					Low active					Fully active				
	n	Death	HR		95% CI	n	Death	HR		95% CI	n	Death	HR		95% CI
**All causes**															
Non-COPD	175,246	2996	1.00		–	74,665	1083	0.90	^a^	(0.8, 1.0)	79,279	1846	0.78	^a^	(0.7, 0.8)
COPD	9203	867	1.40	*	(1.3, 1.5)	4068	302	1.41	*	(1.2, 1.6)	5003	534	1.10		(0.9, 1.2)
**All cancer**															
Non-COPD	175,246	1305	1.00		–	74,665	493	0.94		(0.8, 1.0)	79,279	863	0.85	^a^	(0.8, 0.9)
COPD	9203	280	1.08		(0.9, 1.3)	4068	108	1.23		(1.0, 1.5)	5003	180	0.90		(0.8, 1.1)
**Cardiovascular disease**															
Non-COPD	175,246	506	1.00		–	74,665	175	0.82	^a^	(0.7, 1.0)	79,279	330	0.69	^a^	(0.6, 0.8)
COPD	9203	194	1.50	*	(1.2, 1.8)	4068	54	1.11		(0.8, 1.5)	5003	130	1.19		(1.0, 1.5)
**Respiratory system**															
Non-COPD	175,246	110	1.00		–	74,665	30	0.68		(0.4, 1.0)	79,279	72	0.64	^a^	(0.5, 0.9)
COPD	9203	107	2.79	*	(2.1, 3.8)	4068	37	3.05	*	(2.0, 4.5)	5003	64	2.13	*	(1.5, 3.0)
Expanded CVD															
Non-COPD	175,246	721	1.00		–	74,665	254	0.84	^a^	(0.7, 1.0)	79,279	452	0.68	^a^	(0.6, 0.8)
COPD	9203	284	1.49	*	(1.3, 1.7)	4068	82	1.23		(1.0, 1.6)	5003	179	1.15		(0.9, 1.4)

HRs were adjusted for age, gender, education, BMI, smoking, anemia, hypertension, and blood glucose *Indicates a significantly (*p* < 0.05) higher mortality compared to the reference group. ^a^indicates a significantly (*p* < 0.05) lower mortality compared to the reference group. The reference group is Non-COPD subjects who had inactive LTPA.

For life expectancy estimation (Table 5), the remaining life lost around 5–6 years in both male and female participants with COPD as compared with corresponding non-COPD populations in the same age range. Among the COPD population, participants who were fully active had longer life expectancies than patients who were inactive, an additional 2.4–4.0 years in men and 4.4–4.8 years in women. Notably, the fully active male participants with COPD could have life expectancy similar to that of the male inactive cohort or 1 year less than the overall male cohort. In the female subgroup, fully active participants with COPD could be expected to live even 2 or 1 years longer than the inactive or overall cohorts, respectively.

**Table 5:** Remaining years of life at different ages by COPD and physical activity status.

	Age	Non-COPD^a^	Inactive cohort without COPD^b^	COPD^c^	Inactive COPD^d^	Fully active COPD^e^	Inactive cohort^f^	Cohort^g^	*D*^*c-a*^	*D*^*e-d*^	*D*^*f-e*^	*D*^*g-e*^	*D*^*b-e*^
Male	30	55.86	55.55	49.82	48.21	52.23	52.06	53.29	**(6.0)**	4.0	**(0.2)**	1.1	3.3
	40	46.21	45.91	40.41	39.29	42.86	42.40	43.64	**(5.8)**	3.6	**(0.5)**	0.8	3.1
	50	36.81	36.53	31.14	29.78	33.20	33.03	34.26	**(5.7)**	3.4	**(0.2)**	1.1	3.3
	60	27.99	27.94	22.70	21.38	24.41	24.45	25.55	**(5.3)**	3.0	0.04	1.1	3.5
	70	20.24	20.21	15.24	13.94	16.30	16.92	17.89	**(5.0)**	2.4	0.6	1.6	3.9
Female	30	60.99	57.00	54.98	52.76	57.36	55.38	56.05	**(6.0)**	4.6	**(2.0)**	**(1.3)**	**(0.4)**
	40	51.21	47.19	45.21	43.00	47.77	45.57	46.27	**(6.0)**	4.8	**(2.2)**	**(1.5)**	**(0.6)**
	50	41.61	37.51	35.70	33.30	38.12	35.90	36.67	**(5.9)**	4.8	**(2.2)**	**(1.5)**	**(0.6)**
	60	32.31	28.12	26.73	24.28	28.84	26.59	27.40	**(5.6)**	4.6	**(2.3)**	**(1.4)**	**(0.7)**
	70	23.58	19.28	18.44	15.93	20.36	17.89	18.70	**(5.1)**	4.4	**(2.5)**	**(1.7)**	**(1.1)**

Data in bold means negative value.

## Discussion

The ability of regular physical activity to reduce the harm of COPD has been demonstrated in this study with the number of years gained in life expectancy. On average, participants with COPD lived 6 years less than non-COPD subjects. Female participants with COPD who were fully active achieved an extended life expectancy similar to that of females without COPD, and the entire inactive group in males, implying that the ability of regular exercise to reduce the harms of COPD came close to their actual elimination.

This is the first report of systemic benefits of exercise for patients with COPD in extending their life expectancy. In this study, we found, from cause-specific mortality tables, that the deleterious effects of COPD were devastating and systemic, and not limited to the lungs, as generally perceived by the patients. The relationship of COPD and the lungs is similar to the relationship between chronic kidney disease (CKD) and the kidneys in that the majority of CKD patients die not from kidney disease but from CVD and extra-renal causes^23^. The extra-pulmonary causes of death from COPD accounted for 65% of COPD excess mortality.

To further explain the high rate of extrapulmonary causes of death, we found that nearly one third (31.6%) of subjects with COPD had rapid resting heart rates (≥ 80/min), a 28% increase over the non-COPD group (Table 2), most likely due to obstructed airflow leading to left ventricular diastolic impaired filling^24^. Impaired oxygen exchange with compensatory increase in cardiac output in patients with COPD also leads to heart rate elevation^25^. More severe COPD was associated with a higher likelihood of a rapid resting heart rate in this cohort (Table 2). Our study findings are comparable with those of previous study that reported resting heart rate as a predictor of mortality in patients with COPD^26^. Patients with rapid resting heart rate are known to have higher all-cause mortality^26^, and in this study, even participants within the high normal range (80–99/min) had increased mortality from CVD (both stroke and ischemic heart disease), diabetes, kidney disease and liver diseases (Table A7 in Appendix file).

The rapid resting heart rate might be one of the processes by which COPD transforms from a simple lung disease into a systemic one. Regular exercise can reduce the high normal heart rate of patients with COPD (Table A1) and reverse the systemic effect on CVD death, making exercise effective in patients with COPD. In addition, previous studies have indicated that exercise training can induce important adaptive and beneficial autonomic and cardiovascular adjustments, ensuring proper blood perfusion of peripheral tissues according to metabolic demands^27,28^. Interestingly, our study provides evidence in support of exercise‐induced neuronal plasticity in central autonomic networks^27^, which might be one of the important underlying mechanisms of prolonging life expectancy in patients with COPD. A systemic disease like COPD requires a systemic approach to regular exercise, and meeting the guideline for physical activity would be most beneficial to patients with COPD.

There are several strengths of this study. First, we quantified individuals’ exercise volumes by assigning MET-hours/week to each subject in the cohort, developed from a product of exercise duration (and frequency) and intensity. Although history of regular exercise was self-reported, the data were validated in our previous publications^17,29^. We compared outcomes in individuals who made at least two visits and were consistent in their reporting of exercise volumes. Second, the relationship between heart rate and COPD has been rarely reported^26^, especially the impact of exercise, which is missing even in the GOLD document. Third, the COPD prevalence derived from the cohort of nearly half a million subjects was converted into national prevalence by adjusting for age (5-year intervals) and gender to reflect the actual situation in Taiwan. A similar conversion method was applied in our previous publication on CKD to calculate its corresponding national prevalence^30^. Fourth, we are the first to report the life shortening effects of active and inactive COPD. Life expectancy is derived from combining age-specific mortality rates and is a scientifically valid index. The reason we were able to accomplish the survival analysis was that the life table method required a large number of deaths in each age group for age-specific mortality rates to be stable. Some studies used modeling to arrive at that because of inadequate sample sizes. In addition, the use of life expectancy made the results easily understandable and could motivate patients with COPD to exercise. Fifth, the health surveillance of half a million subjects by the MJ Health Management Institution, including lung function and blood tests, was standardized with identical instruments and interpretations. This minimized the variations encountered in other studies.

There are several important limitations. First, the effect of regular exercise on patients with COPD that we observed came from statistical associations and not clinical trials, so causal interpretation should be avoided. Healthier patients with COPD were able to exercise more and showed lower mortality. The question arose as to which came first, whether exercisers become healthier first or healthier people tended to do more exercise. The opposite is also true for a vicious circle in that the more inactive the people, the worse the COPD, and vice versa. It is clear that exercise is additive and cumulative, and regardless of causality, more exercise would definitely lead to better outcomes for patients with COPD, whether at stage 1 or stage 4. However, there were several clues pointing to causality according to Bradford Hill’s criteria: The dose response relationship, life expectancy results collaborating HR results, exercise effect on heart rate, on diabetes, and on kidney diseases available in the literature, and consistency between males and females, as well as between smokers and nonsmokers. Second, the definition of COPD in the present study was not based on post-bronchodilator data, as suggested by the GOLD guidelines. This requirement is usually sufficient for defining COPD in epidemiological studies. Without clinical confirmation of symptoms and signs, the number of patients with COPD identified would obviously be overestimated. On the other hand, the definition used in epidemiological studies, based on spirometry, correlated well with mortality outcomes and served the purpose of taking preventive action against COPD. Third, the self-paid nature of health surveillance at the MJ Institution would attract individuals of higher socioeconomic class, so selection bias was possible. Thus, the patients with COPD in the cohort may not be representative of society. However, given the large sample size and inclusion of extended family members, the bias may be minimal. Fourth, the results were based on initial assessments of lung function and did not consider subsequent courses of disease or development. Many subjects, up to half in each round, returned for up to 9 rounds, within an average span of 18 months between visits^18^. What we demonstrated, however, was the power of the initial visit in predicting the long-term outcome. We worked on data from the second visit and found the outcome results of COPD at second visits nearly identical (Table A4 in the Appendix file). Fifth, the evolution of COPD therapy might have influenced this cohort during the long follow up. Because the proportion of deaths caused by COPD among the all-cause mortality was similar in every year during the follow up, the influence on mortality might not be significant. Last, the information on physical activity was collected based on three multiple choice questions, so recall bias might exist.

In conclusion, COPD is a prevalent disease in Asian countries and impacts survival in all stages. Around 65% of deaths related to COPD are attributed to extra-pulmonary disease and the increased heart rate caused by COPD, indicating systemic involvement. The abbreviation of life expectancy of around 6 years by COPD can be mostly reversed by fully active exercise, especially in female patients. Prescribing regular exercise to patients with COPD is quite important and needs to be incorporated into the care bundle.

## Supplementary Information

Supplementary Information.
