# Supplementary material for: The ability of physical activity in reducing mortality risks and cardiovascular loading and in extending life expectancy in patients with COPD
Source: Sci Rep. 2021 Nov 4;11:21674. doi: 10.1038/s41598-021-00728-2 (PMC8569178; doi:10.1038/s41598-021-00728-2)
Supplement: Supplementary file 1 — Supplementary Information. [file 41598_2021_728_MOESM1_ESM.docx]

*Appendix file*

**Title:** The ability of physical activity in reducing mortality risks and cardiovascular loading and in extending life expectancy in patients with COPD

**Authors:** Chin-Chung Shu^1*^, June Han Lee^2*^, Min Kwang Tsai^2^, Ta-Chen Su^1,3#^, and Chi Pang Wen^2#^

^1^Department of Internal Medicine, National Taiwan University Hospital, Taipei, Taiwan

^2^ National Health Research Institutes, Taiwan

^3^Environmental and Occupational Medicine, National Taiwan University Hospital, Taipei, Taiwan

*equal contribution

^#^Corresponding authors

**Correspondence:**

Chi Pang Wen

National Health Research Institutes, Taiwan

Email: [cwengood@nhri.edu.tw](mailto:cwengood@nhri.edu.tw)

Ta-Chen Su

Environmental and Occupational Medicine, National Taiwan University Hospital, Taipei, Taiwan

[tachensu@gmail.com](mailto:tachensu@gmail.com)

Running Title: Exercise on COPD life expectancy

**Table A1.** Estimated additional deaths result from COPD for all participants.

| **Cause of death** | **Non-COPD** | |  | **COPD** | | | | All deaths | Additional death (count) | % among total additional death | Additional death for extra pulmonary disease % |
| --- | --- | --- | --- | --- | --- | --- | --- | --- | --- | --- | --- |
|  | Death | HR |  | Death | HR | 95% CI | |  |  |  |  |
| **All Causes** | 8,458 | 1.00 |  | 3,531 | **1.44** | (1.35 | , 1.53) | 11,989 | 1074 | 100% | 65% |
| **All Cancer** | 3,666 | 1.00 |  | 1134 | **1.11** | (1.01 | , 1.23) | 4,800 | 116 | 11% |  |
| Lung cancer | 688 | 1.00 |  | 301 | **1.42** | (1.16 | , 1.72) | 989 | 88 | 8% |  |
| Non-lung cancer | 2,978 | 1.00 |  | 833 | 1.03 | (0.92 | , 1.16) | 3811 |  |  |  |
| **Cardiovascular disease** | 1495 | 1.00 |  | 781 | **1.55** | (1.36 | , 1.77) | 2,276 | 278 | 26% |  |
| Ischemic heart disease | 421 | 1.00 |  | 208 | **1.58** | (1.24 | , 2.02) | 629 | 77 | 7% |  |
| Stroke | 620 | 1.00 |  | 332 | **1.55** | (1.26 | , 1.91) | 952 | 118 | 11% |  |
| **Infectious disease** | 100 | 1.00 |  | 72 | **1.97** | (1.21 | , 3.21) | 172 | 35 | 3% |  |
| **Respiratory system** | 326 | 1.00 |  | 424 | **3.22** | (2.58 | , 4.00) | 750 | 292 | 27% |  |
| COPD | 68 | 1.00 |  | 242 | **11.78** | (7.75 | , 17.90) | 310 | 221 | 21% |  |
| Pneumonia | 165 | 1.00 |  | 119 | **1.74** | (1.22 | , 2.48) | 284 | 50 | 5% |  |
| **Digestive system** | 578 | 1.00 |  | 221 | **1.45** | (1.15 | , 1.84) | 799 | 69 | 6% |  |
| **Genitourinary system** | 229 | 1.00 |  | 116 | **1.57** | (1.12 | , 2.21) | 345 | 42 | 4% |  |
| Kidney diseases | 185 | 1.00 |  | 99 | **1.82** | (1.25 | , 2.64) | 284 | 44 | 4% |  |
| **Diabetes mellitus** | 461 | 1.00 |  | 259 | **5.37** | (4.32 | , 6.68) | 720 | 211 | 20% |  |
| **Expanded CVD** | 2141 | 1.00 |  | 1139 | **1.55** | (1.38 | , 1.73) | 3,280 | 402 | 37% |  |

HRs were adjusted for age, gender, education, BMI, smoking, physical activity, anemia, hypertension and blood glucose.

*p*-values smaller than 0.05 are indicated in bold.

**Table A2a.** Male mortality risk for different chronic obstructive pulmonary disease stages by cause of death

| **Cause of death** | **Non-COPD** | |  | **COPD** | |  | **COPD** | | | | | | | | | | | |
| --- | --- | --- | --- | --- | --- | --- | --- | --- | --- | --- | --- | --- | --- | --- | --- | --- | --- | --- |
|  |  |  |  |  |  |  | **Stage 1** | |  | **Stage 2** | |  | **Stage 3** | |  | **Stage 4** | |  |
|  | Death | HR |  | Death | HR |  | Death | HR |  | Death | HR |  | Death | HR |  | Death | HR |  |
| **All Causes** | 5,134 | 1.00 |  | 2,359 | **1.46** | ***** | 448 | 1.12 |  | 1124 | **1.45** | ***** | 561 | **1.75** | ***** | 226 | **2.65** | ***** |
| **All Cancer** | 2,166 | 1.00 |  | 786 | **1.14** | ***** | 164 | 0.92 |  | 410 | **1.28** | ***** | 172 | **1.27** | ***** | 40 | 1.08 |  |
| Lung cancer | 428 | 1.00 |  | 235 | **1.54** | ***** | 50 | 1.39 |  | 119 | **1.62** | ***** | 54 | **1.76** | ***** | 12 | 1.40 |  |
| Non Lung cancer | 1,738 | 1.00 |  | 551 | **1.02** |  | 114 | 0.77 |  | 291 | 1.19 |  | 118 | 1.13 |  | 28 | 0.96 |  |
| **Cardiovascular disease** | 934 | 1.00 |  | 508 | **1.58** | ***** | 103 | 1.24 |  | 245 | **1.63** | ***** | 111 | **1.53** | ***** | 49 | **3.46** | ***** |
| Ischemic heart disease | 284 | 1.00 |  | 155 | **1.77** | ***** | 28 | 1.24 |  | 72 | **1.79** | ***** | 36 | **1.77** | ***** | 19 | **4.21** | ***** |
| Stroke | 379 | 1.00 |  | 219 | **1.68** | ***** | 41 | 1.28 |  | 114 | **1.84** | ***** | 45 | **1.73** | ***** | 19 | **3.59** | ***** |
| **Infectious disease** | 63 | 1.00 |  | 53 | **1.96** | ***** | 9 | 1.35 |  | 20 | 1.21 |  | 18 | **4.56** | ***** | 6 | **4.97** | ***** |
| **Respiratory system** | 223 | 1.00 |  | 327 | **3.76** | ***** | 36 | 1.52 |  | 113 | **2.87** | ***** | 106 | **6.43** | ***** | 72 | **12.70** | ***** |
| COPD | 44 | 1.00 |  | 192 | **16.35** | ***** | 15 | **4.66** | ***** | 56 | **9.47** | ***** | 68 | **28.20** | ***** | 53 | **71.02** | ***** |
| Pneumonia | 117 | 1.00 |  | 88 | **1.99** | ***** | 16 | 1.37 |  | 36 | **2.01** | ***** | 21 | **2.21** | ***** | 15 | **5.29** | ***** |
| **Digestive system** | 358 | 1.00 |  | 143 | **1.48** | ***** | 28 | 1.24 |  | 77 | **1.84** | ***** | 25 | 1.08 |  | 13 | **2.75** | ***** |
| **Genitourinary system** | 120 | 1.00 |  | 63 | 1.40 |  | 12 | 0.80 |  | 24 | 0.86 |  | 18 | **2.45** | ***** | 9 | **6.91** | ***** |
| Kidney diseases | 99 | 1.00 |  | 55 | 1.59 |  | 10 | 0.81 |  | 20 | 0.96 |  | 17 | **2.83** | ***** | 8 | **7.77** | ***** |
| **Diabetes mellitus** | 245 | 1.00 |  | 139 | **1.44** | ***** | 31 | 1.33 |  | 64 | **1.58** | ***** | 29 | 1.79 |  | 15 | 1.29 |  |
| **Expanded CVD** | 1278 | 1.00 |  | 702 | **1.56** | ***** | 144 | 1.22 |  | 329 | **1.57** | ***** | 157 | **1.68** | ***** | 72 | **3.31** | ***** |
| **Nonsmoker** |  |  |  |  |  |  |  |  |  |  |  |  |  |  |  |  |  |  |
| **All Causes** | 1,170 | 1.00 |  | 276 | **1.53** | ***** | 67 | 1.20 |  | 133 | **1.57** | ***** | 53 | **1.70** | ***** | 23 | **3.05** | ***** |
| **All Cancer** | 485 | 1.00 |  | 98 | **1.33** | ***** | 23 | 1.03 |  | 52 | **1.54** | ***** | 17 | 1.40 |  | 6 | 2.06 |  |
| Lung cancer | 69 | 1.00 |  | 22 | **2.19** | ***** | 5 | 1.56 |  | 9 | 1.86 |  | 5 | **3.08** | ***** | 3 | **6.75** | ***** |
| Non Lung cancer | 416 | 1.00 |  | 76 | 1.19 |  | 18 | 1.01 |  | 43 | **1.24** | ***** | 12 | **1.31** | ***** | 3 | **1.25** | ***** |
| **Cardiovascular disease** | 205 | 1.00 |  | 62 | **1.79** | ***** | 17 | 1.53 |  | 31 | **1.91** | ***** | 11 | 1.83 |  | 3 | 1.97 |  |
| Ischemic heart disease | 60 | 1.00 |  | 23 | **2.38** | ***** | 5 | 1.65 |  | 13 | **2.73** | ***** | 5 | **3.07** | ***** | 0 | 0.00 |  |
| Stroke | 70 | 1.00 |  | 26 | **1.99** | ***** | 8 | 2.00 |  | 13 | **2.31** | ***** | 3 | 1.37 |  | 2 | 3.78 |  |
| **Infectious disease** | 13 | 1.00 |  | 4 | 1.52 |  | 0 | 0.00 |  | 2 | 1.62 |  | 1 | 2.02 |  | 1 | **9.82** | ***** |
| **Respiratory system** | 39 | 1.00 |  | 34 | **3.34** | ***** | 8 | 2.08 |  | 11 | **2.74** | ***** | 8 | **4.03** | ***** | 7 | **19.54** | ***** |
| COPD | 1 | 1.00 |  | 16 | **-** |  | 3 | **35.93** | ***** | 3 | **22.01** | ***** | 5 | **91.24** | ***** | 5 | **574.92** | ***** |
| Pneumonia | 24 | 1.00 |  | 11 | 1.70 |  | 3 | 1.14 |  | 5 | 2.21 |  | 1 | 0.92 |  | 2 | **8.54** | ***** |
| **Digestive system** | 70 | 1.00 |  | 23 | **2.08** | ***** | 6 | 1.97 |  | 11 | **2.28** | ***** | 4 | 2.23 |  | 2 | **5.03** | ***** |
| **Genitourinary system** | 33 | 1.00 |  | 11 | 1.54 |  | 1 | 0.40 |  | 5 | 1.64 |  | 3 | 2.38 |  | 2 | **8.43** | ***** |
| Kidney diseases | 27 | 1.00 |  | 10 | 1.82 |  | 1 | 0.50 |  | 5 | 2.04 |  | 3 | 3.16 |  | 1 | 5.17 |  |
| **Diabetes mellitus** | 55 | 1.00 |  | 13 | 1.06 |  | 5 | 1.30 |  | 7 | 1.26 |  | 1 | 0.73 |  | 0 | 0.00 |  |
| **Expanded CVD** | 287 | 1.00 |  | 85 | **1.65** | ***** | 23 | 1.36 |  | 43 | **1.79** | ***** | 15 | **1.74** | ***** | 4 | 1.79 |  |
| **Smoker** |  |  |  |  |  |  |  |  |  |  |  |  |  |  |  |  |  |  |
| **All Causes** | 2,273 | 1.00 |  | 818 | **1.47** | ***** | 157 | 1.09 |  | 374 | **1.42** | ***** | 204 | **1.78** | ***** | 83 | **2.59** | ***** |
| **All Cancer** | 1,043 | 1.00 |  | 276 | 1.10 |  | 57 | 0.90 |  | 144 | **1.23** | ***** | 63 | 1.26 |  | 12 | 0.89 |  |
| Lung cancer | 248 | 1.00 |  | 99 | **1.46** | ***** | 25 | 1.39 |  | 50 | **1.61** | ***** | 21 | **1.62** | ***** | 3 | 0.78 |  |
| Non Lung cancer | 795 | 1.00 |  | 177 | 0.97 |  | 32 | 0.71 |  | 94 | 1.10 |  | 42 | 1.14 |  | 9 | 0.92 |  |
| **Cardiovascular disease** | 397 | 1.00 |  | 171 | **1.52** | ***** | 35 | 1.13 |  | 78 | **1.53** | ***** | 33 | 1.44 |  | 25 | **3.84** | ***** |
| Ischemic heart disease | 126 | 1.00 |  | 57 | **1.58** | ***** | 12 | 1.09 |  | 23 | 1.48 |  | 11 | 1.40 |  | 11 | **4.83** | ***** |
| Stroke | 153 | 1.00 |  | 71 | **1.63** | ***** | 11 | 1.01 |  | 35 | **1.70** | ***** | 16 | **1.81** | ***** | 9 | **3.57** | ***** |
| **Infectious disease** | 24 | 1.00 |  | 20 | **2.14** | ***** | 5 | 1.90 |  | 4 | 1.08 |  | 9 | **5.26** | ***** | 2 | 3.93 |  |
| **Respiratory system** | 87 | 1.00 |  | 126 | **3.94** | ***** | 13 | 1.25 |  | 40 | **2.86** | ***** | 47 | **6.91** | ***** | 26 | **11.52** | ***** |
| COPD | 17 | 1.00 |  | 77 | **13.08** | ***** | 6 | **2.89** | ***** | 21 | **7.90** | ***** | 30 | **22.93** | ***** | 20 | **48.00** | ***** |
| Pneumonia | 41 | 1.00 |  | 34 | **2.15** | ***** | 7 | 1.42 |  | 13 | **1.91** | ***** | 9 | **2.62** | ***** | 5 | **4.48** | ***** |
| **Digestive system** | 150 | 1.00 |  | 44 | 1.30 |  | 7 | 0.95 |  | 27 | **1.68** | ***** | 5 | 0.75 |  | 5 | 2.18 |  |
| **Genitourinary system** | 43 | 1.00 |  | 19 | 1.30 |  | 4 | 1.01 |  | 3 | 0.46 |  | 7 | **2.41** | ***** | 5 | **5.98** | ***** |
| Kidney diseases | 33 | 1.00 |  | 16 | 1.45 |  | 3 | 0.98 |  | 2 | 0.39 |  | 6 | **2.61** | ***** | 5 | **7.96** | ***** |
| **Diabetes mellitus** | 100 | 1.00 |  | 52 | **1.66** | ***** | 9 | 1.41 |  | 26 | **1.77** | ***** | 14 | **2.28** | ***** | 3 | 1.86 |  |
| **Expanded CVD** | 530 | 1.00 |  | 239 | **1.53** | ***** | 47 | 1.17 |  | 106 | **1.50** | ***** | 53 | **1.68** | ***** | 33 | **3.78** | ***** |

**Table A2b.** Female mortality risk for different chronic obstructive pulmonary disease stages by cause of death

| **Cause of death** | **Non-COPD** | |  | **COPD** | |  | **COPD** | | | | | | | | | | | | | | | | | | | | |  |
| --- | --- | --- | --- | --- | --- | --- | --- | --- | --- | --- | --- | --- | --- | --- | --- | --- | --- | --- | --- | --- | --- | --- | --- | --- | --- | --- | --- | --- |
|  |  |  |  |  |  |  | **Stage 1** | |  | | **Stage 2** | | | |  | | **Stage 3** | | | |  | | **Stage 4** | | | |  |  |
|  | Death | HR |  | Death | HR |  | Death | HR |  | | Death | | HR | |  | | Death | | HR | |  | | Death | | HR | |  |  |
| **All Causes** | 3,324 | 1.00 |  | 1,172 | **1.38** | ***** | 243 | 1.22 |  | | 554 | | **1.30** | | ***** | | 270 | | **1.57** | | ***** | | 105 | | **2.08** | | ***** |  |
| **All Cancer** | 1,500 | 1.00 |  | 348 | 1.07 |  | 75 | 0.97 |  | | 175 | | 1.14 | |  | | 77 | | 1.15 | |  | | 21 | | 1.25 | |  |  |
| Lung cancer | 260 | 1.00 |  | 66 | 1.08 |  | 10 | 0.80 |  | | 29 | | 1.16 | |  | | 21 | | 1.29 | |  | | 6 | | 1.67 | |  |  |
| Non Lung cancer | 1,240 | 1.00 |  | 282 | 1.07 |  | 65 | 1.02 |  | | 146 | | 1.15 | |  | | 56 | | 1.12 | |  | | 15 | | 1.17 | |  |  |
| **Cardiovascular disease** | 561 | 1.00 |  | 273 | **1.50** | ***** | 55 | 1.22 |  | | 120 | | 1.15 | |  | | 67 | | **2.25** | | ***** | | 31 | | **3.42** | | ***** |  |
| Ischemic heart disease | 137 | 1.00 |  | 53 | 1.07 |  | 11 | 0.67 |  | | 19 | | 0.80 | |  | | 16 | | 1.95 | |  | | 7 | | **3.53** | | ***** |  |
| Stroke | 241 | 1.00 |  | 113 | 1.32 |  | 26 | 1.73 |  | | 51 | | 1.06 | |  | | 25 | | 1.12 | |  | | 11 | | 2.32 | |  |  |
| **Infectious disease** | 37 | 1.00 |  | 19 | 2.02 |  | 8 | 2.82 |  | | 3 | | 0.00 | |  | | 7 | | 4.11 | |  | | 1 | | 6.13 | |  |  |
| **Respiratory system** | 103 | 1.00 |  | 97 | **1.78** | ***** | 17 | 1.99 |  | | 38 | | 1.07 | |  | | 31 | | **2.80** | | ***** | | 11 | | **4.55** | | ***** |  |
| COPD | 24 | 1.00 |  | 50 | **5.07** | ***** | 6 | **4.83** | ***** | | 10 | | 1.26 | |  | | 24 | | **10.82** | | ***** | | 10 | | **16.42** | | ***** |  |
| Pneumonia | 48 | 1.00 |  | 31 | 1.04 |  | 6 | 0.79 |  | | 19 | | 1.35 | |  | | 5 | | 0.86 | |  | | 1 | | 2.20 | |  |  |
| **Digestive system** | 220 | 1.00 |  | 78 | 1.40 |  | 15 | 1.05 |  | | 39 | | 1.22 | |  | | 10 | | 1.33 | |  | | 14 | | **3.80** | | ***** |  |
| **Genitourinary system** | 109 | 1.00 |  | 53 | **1.93** | ***** | 13 | **2.55** | ***** | | 27 | | 1.79 | |  | | 10 | | 2.20 | |  | | 3 | | 1.43 | |  |  |
| Kidney diseases | 86 | 1.00 |  | 44 | **2.31** | ***** | 12 | **3.52** | ***** | | 22 | | **2.20** | | ***** | | 8 | | 1.50 | |  | | 2 | | 2.02 | |  |  |
| **Diabetes mellitus** | 216 | 1.00 |  | 120 | 1.46 |  | 21 | 1.67 |  | | 61 | | **2.02** | | ***** | | 31 | | **4.84** | | ***** | | 7 | | 0.19 | |  |  |
| **Expanded CVD** | 863 | 1.00 |  | 437 | **1.52** | ***** | 88 | 1.34 |  | | 203 | | **1.33** | | ***** | | 106 | | **2.20** | | ***** | | 40 | | **2.44** | | ***** |  |
| **Nonsmoker** |  |  |  |  |  |  |  |  |  | |  | |  | |  | |  | |  | |  | |  | |  | |  |  |
| **All Causes** | 1,777 | 1.00 |  | 383 | **1.39** | ***** | 85 | 1.20 |  | | 175 | | **1.31** | | ***** | | 78 | | **1.55** | | ***** | | 45 | | **2.17** | | ***** |  |
| **All Cancer** | 885 | 1.00 |  | 132 | 1.06 |  | 27 | 0.92 |  | | 70 | | 1.18 | |  | | 24 | | 1.09 | |  | | 11 | | 1.25 | |  |  |
| Lung cancer | 147 | 1.00 |  | 22 | 0.99 |  | 4 | 0.78 |  | | 13 | | 1.24 | |  | | 3 | | 0.79 | |  | | 2 | | 1.26 | |  |  |
| Non Lung cancer | 738 | 1.00 |  | 110 | 1.08 |  | 23 | 1.01 |  | | 57 | | **1.24** | | ***** | | 21 | | **1.31** | | ***** | | 9 | | 1.25 | |  |  |
| **Cardiovascular disease** | 270 | 1.00 |  | 87 | **1.58** | ***** | 21 | 1.37 |  | | 29 | | 1.17 | |  | | 21 | | **2.25** | | ***** | | 16 | | **3.77** | | ***** |  |
| Ischemic heart disease | 71 | 1.00 |  | 15 | 0.94 |  | 3 | 0.68 |  | | 4 | | 0.58 | |  | | 4 | | 1.57 | |  | | 4 | | **3.23** | | ***** |  |
| Stroke | 116 | 1.00 |  | 36 | **1.52** | ***** | 13 | **1.95** | ***** | | 13 | | 1.23 | |  | | 5 | | 1.25 | |  | | 5 | | **2.75** | | ***** |  |
| **Infectious disease** | 13 | 1.00 |  | 5 | 2.69 |  | 2 | 4.05 |  | | 0 | | 0.00 | |  | | 2 | | **6.12** | | ***** | | 1 | | 7.84 | |  |  |
| **Respiratory system** | 54 | 1.00 |  | 26 | **1.98** | ***** | 8 | **2.22** | ***** | | 7 | | 1.17 | |  | | 7 | | **2.98** | | ***** | | 4 | | **5.25** | | ***** |  |
| COPD | 12 | 1.00 |  | 15 | **6.53** | ***** | 4 | **6.34** | ***** | | 2 | | 1.83 | |  | | 6 | | **12.58** | | ***** | | 3 | | **21.21** | | ***** |  |
| Pneumonia | 25 | 1.00 |  | 8 | 1.06 |  | 1 | 0.50 |  | | 5 | | 1.56 | |  | | 1 | | 1.03 | |  | | 1 | | 2.45 | |  |  |
| **Digestive system** | 112 | 1.00 |  | 29 | 0.34 |  | 5 | 0.90 |  | | 13 | | 1.24 | |  | | 5 | | 1.41 | |  | | 6 | | **3.74** | | ***** |  |
| **Genitourinary system** | 45 | 1.00 |  | 18 | **1.96** | ***** | 7 | **2.79** | ***** | | 7 | | 1.69 | |  | | 4 | | **2.84** | | ***** | | 0 | | 0.00 | |  |  |
| Kidney diseases | 35 | 1.00 |  | 15 | **2.23** | ***** | 7 | **3.87** | ***** | | 6 | | 1.98 | |  | | 2 | | 1.92 | |  | | 0 | | 0.00 | |  |  |
| **Diabetes mellitus** | 106 | 1.00 |  | 28 | 1.25 |  | 4 | 0.60 |  | | 16 | | 1.72 | |  | | 7 | | **2.73** | | ***** | | 1 | | 0.24 | |  |  |
| **Expanded CVD** | 411 | 1.00 |  | 130 | **1.49** | ***** | 32 | 1.35 |  | | 51 | | 1.28 | |  | | 30 | | **2.12** | | ***** | | 17 | | **2.51** | | ***** |  |
| **Smoker** |  |  |  |  |  |  |  |  |  | |  | |  | |  | |  | |  | |  | |  | |  | |  |  |
| **All Causes** | 220 | 1.00 |  | 68 | **1.36** | ***** | 18 | 1.38 |  | | 27 | | 1.23 | |  | | 19 | | **1.72** | | ***** | | 4 | | 1.39 | |  |  |
| **All Cancer** | 68 | 1.00 |  | 16 | 1.15 |  | 5 | **1.52** | ***** | | 5 | | 0.88 | |  | | 5 | | 1.68 | |  | | 1 | | 1.35 | |  |  |
| Lung cancer | 13 | 1.00 |  | 6 | 1.57 |  | 1 | 0.66 |  | | 1 | | 0.67 | |  | | 3 | | **4.04** | | ***** | | 1 | | 5.23 | |  |  |
| Non Lung cancer | 55 | 1.00 |  | 10 | 1.02 |  | 4 | 1.97 |  | | 4 | | 0.97 | |  | | 2 | | 0.92 | |  | | 0 | | 0.00 | |  |  |
| **Cardiovascular disease** | 44 | 1.00 |  | 18 | 1.20 |  | 3 | 0.68 |  | | 7 | | 0.95 | |  | | 7 | | 2.24 | |  | | 1 | | 1.36 | |  |  |
| Ischemic heart disease | 11 | 1.00 |  | 19 | 1.95 |  | 1 | 0.71 |  | | 3 | | 1.35 | |  | | 3 | | 3.20 | |  | | 1 | | 4.90 | |  |  |
| Stroke | 19 | 1.00 |  | 4 | 0.47 |  | 2 | 0.84 |  | | 1 | | 0.27 | |  | | 1 | | 0.71 | |  | | 0 | | 0.00 | |  |  |
| **Infectious disease** | 3 | 1.00 |  | 0 | - |  | 0 | 0.00 |  | | 0 | | 0.00 | |  | | 0 | | 0.00 | |  | | 0 | | 0.00 | |  |  |
| **Respiratory system** | 8 | 1.00 |  | 3 | 0.87 |  | 1 | 1.04 |  | | 1 | | 1.02 | |  | | 1 | | 1.85 | |  | | 0 | | 0.00 | |  |  |
| COPD | 3 | 1.00 |  | 1 | 0.40 |  | 0 | 0.00 |  | | 0 | | 0.00 | |  | | 1 | | 3.95 | |  | | 0 | | 0.00 | |  |  |
| Pneumonia | 3 | 1.00 |  | 1 | 0.64 |  | 1 | 1.89 |  | | 0 | | 0.00 | |  | | 0 | | 0.00 | |  | | 0 | | 0.00 | |  |  |
| **Digestive system** | 16 | 1.00 |  | 6 | 1.60 |  | 2 | 1.80 |  | | 2 | | 1.08 | |  | | 1 | | 1.18 | |  | | 1 | | 4.23 | |  |  |
| **Genitourinary system** | 7 | 1.00 |  | 4 | 1.68 |  | 1 | 1.25 |  | 2 | | 2.18 | |  | | 0 | | 0.00 | |  | | 1 | | **8.82** | | ***** | | |
| Kidney diseases | 5 | 1.00 |  | 4 | 2.46 |  | 1 | 1.92 |  | 2 | | 2.97 | |  | | 0 | | 0.00 | |  | | 1 | | **13.62** | | ***** | | |
| **Diabetes mellitus** | 10 | 1.00 |  | 9 | **3.39** | ***** | 3 | 3.52 |  | 3 | | 3.28 | |  | | 3 | | **5.30** | | ***** | | 0 | | 0.00 | |  | | |
| **Expanded CVD** | 59 | 1.00 |  | 31 | **1.71** | ***** | 7 | 1.27 |  | 12 | | 1.52 | |  | | 10 | | **2.52** | | ***** | | 2 | | 1.86 | |  | | |

**Table A3.** Mortality risk for different chronic obstructive pulmonary disease and smoking status

| **Cause of death** | **Non-COPD** | | | | | | **COPD** | | | | | |
| --- | --- | --- | --- | --- | --- | --- | --- | --- | --- | --- | --- | --- |
|  | **Non-smoker** | |  | **Smoker** | | | **Non-smoker** | | | **Smoker** | | |
|  | Death | HR |  | Death | HR |  | Death | HR |  | Death | HR |  |
| **All** |  |  |  |  |  |  |  |  |  |  |  |  |
| **All Causes** | 2,947 | 1.00 |  | 2,493 | **1.56** | ***** | 659 | **1.50** | ***** | 886 | **2.19** | ***** |
| **All Cancer** | 1,370 | 1.00 |  | 1,111 | **1.54** | ***** | 230 | **1.17** | ***** | 292 | **1.67** | ***** |
| Lung cancer | 216 | 1.00 |  | 261 | **2.26** | ***** | 44 | 1.30 |  | 105 | **3.43** | ***** |
| Non-lung cancer | 1154 | 1.00 |  | 850 | **1.41** | ***** | 186 | 1.15 |  | 187 | **1.32** | ***** |
| **Cardiovascular disease** | 475 | 1.00 |  | 441 | **1.67** | ***** | 149 | **1.77** | ***** | 189 | **2.37** | ***** |
| Ischemic heart disease | 131 | 1.00 |  | 137 | **1.69** | ***** | 38 | **1.63** | ***** | 65 | **2.66** | ***** |
| Stroke | 186 | 1.00 |  | 172 | **1.74** | ***** | 62 | **1.81** | ***** | 75 | **2.43** | ***** |
| **Infectious disease** | 26 | 1.00 |  | 27 | 1.56 |  | 9 | 1.88 |  | 20 | **3.19** | ***** |
| **Respiratory system** | 93 | 1.00 |  | 95 | **1.47** | ***** | 60 | **2.83** | ***** | 129 | **5.12** | ***** |
| COPD | 13 | 1.00 |  | 20 | **2.55** | ***** | 31 | **11.95** | ***** | 78 | **28.84** | ***** |
| Pneumonia | 49 | 1.00 |  | 44 | 1.24 |  | 19 | 1.60 |  | 35 | **2.30** | ***** |
| **Digestive system** | 182 | 1.00 |  | 166 | **1.70** | ***** | 52 | **1.86** | ***** | 50 | **2.01** | ***** |
| **Genitourinary system** | 78 | 1.00 |  | 50 | 1.27 |  | 29 | **1.80** | ***** | 23 | 1.68 |  |
| Kidney diseases | 62 | 1.00 |  | 38 | 1.23 |  | 25 | **2.06** | ***** | 20 | **1.90** | ***** |
| **Diabetes mellitus** | 161 | 1.00 |  | 110 | **1.42** | ***** | 41 | 1.21 |  | 61 | **2.46** | ***** |
| **Expanded CVD** | 698 | 1.00 |  | 589 | **1.58** | ***** | 215 | **1.66** | ***** | 270 | **2.33** | ***** |
| **Male** |  |  |  |  |  |  |  |  |  |  |  |  |
| **All Causes** | 1,170 | 1.00 |  | 2,273 | **1.50** | ***** | 276 | **1.59** | ***** | 818 | **2.17** | ***** |
| **All Cancer** | 485 | 1.00 |  | 1,043 | **1.66** | ***** | 98 | **1.36** | ***** | 276 | **1.79** | ***** |
| Lung cancer | 69 | 1.00 |  | 248 | **2.79** | ***** | 22 | **1.97** | ***** | 99 | **4.20** | ***** |
| Non-lung cancer | 416 | 1.00 |  | 795 | **1.48** | ***** | 76 | 1.26 |  | 177 | **1.39** | ***** |
| **Cardiovascular disease** | 205 | 1.00 |  | 397 | **1.52** | ***** | 62 | **1.81** | ***** | 171 | **2.31** | ***** |
| Ischemic heart disease | 60 | 1.00 |  | 126 | **1.62** | ***** | 23 | **2.31** | ***** | 57 | **2.60** | ***** |
| Stroke | 70 | 1.00 |  | 153 | **1.68** | ***** | 26 | **2.14** | ***** | 71 | **2.64** | ***** |
| **Infectious disease** | 13 | 1.00 |  | 24 | 1.24 |  | 4 | 1.44 |  | 20 | **2.70** | ***** |
| **Respiratory system** | 39 | 1.00 |  | 87 | **1.66** | ***** | 34 | **3.69** | ***** | 126 | **6.27** | ***** |
| COPD | 1 | 1.00 |  | 17 | **12.82** | ***** | 16 | **70.58** | ***** | 77 | **167.92** | ***** |
| Pneumonia | 24 | 1.00 |  | 41 | 1.28 |  | 11 | 1.95 |  | 34 | **2.58** | ***** |
| **Digestive system** | 70 | 1.00 |  | 150 | **1.60** | ***** | 23 | **2.27** | ***** | 44 | **1.99** | ***** |
| **Genitourinary system** | 33 | 1.00 |  | 43 | 0.98 |  | 11 | 1.55 |  | 19 | 1.30 |  |
| Kidney diseases | 27 | 1.00 |  | 33 | 0.95 |  | 10 | 1.79 |  | 16 | 1.41 |  |
| **Diabetes mellitus** | 55 | 1.00 |  | 100 | 1.37 |  | 13 | 1.28 |  | 52 | **2.21** | ***** |
| **Expanded CVD** | 287 | 1.00 |  | 530 | **1.43** | ***** | 85 | **1.70** | ***** | 239 | **2.19** | ***** |
| **Female** |  |  |  |  |  |  |  |  |  |  |  |  |
| **All causes** | 1,777 | 1.00 |  | 220 | **1.90** | ***** | 383 | **1.40** | ***** | 68 | **2.44** | ***** |
| **All cancer** | 885 | 1.00 |  | 68 | 1.18 |  | 132 | 1.07 |  | 16 | 1.28 |  |
| Lung cancer | 147 | 1.00 |  | 13 | 1.40 |  | 22 | 0.98 |  | 6 | **2.48** | ***** |
| Non-lung cancer | 738 | 1.00 |  | 55 | 1.13 |  | 110 | 1.09 |  | 10 | 1.01 |  |
| **Cardiovascular disease** | 270 | 1.00 |  | 44 | **2.55** | ***** | 87 | **1.60** | ***** | 18 | **2.74** | ***** |
| Ischemic heart disease | 71 | 1.00 |  | 11 | **2.39** | ***** | 15 | 0.98 |  | 8 | **4.03** | ***** |
| Stroke | 116 | 1.00 |  | 19 | **2.64** | ***** | 36 | **1.52** | ***** | 4 | 1.38 |  |
| **Infectious disease** | 13 | 1.00 |  | 3 | **4.26** | ***** | 5 | 2.70 |  | 0 | 0.00 |  |
| **Respiratory system** | 54 | 1.00 |  | 8 | 1.98 |  | 26 | **2.04** | ***** | 3 | 1.26 |  |
| COPD | 12 | 1.00 |  | 3 | 3.29 |  | 15 | **6.37** | ***** | 1 | 2.60 |  |
| Pneumonia | 25 | 1.00 |  | 3 | 1.53 |  | 8 | 1.16 |  | 1 | 0.64 |  |
| **Digestive system** | 112 | 1.00 |  | 16 | **2.38** | ***** | 29 | 1.42 |  | 6 | **3.12** | ***** |
| **Genitourinary system** | 45 | 1.00 |  | 7 | **2.49** | ***** | 18 | **1.96** | ***** | 4 | **4.86** | ***** |
| Kidney diseases | 35 | 1.00 |  | 5 | 2.34 |  | 15 | **2.23** | ***** | 4 | **6.97** | ***** |
| **Diabetes mellitus** | 106 | 1.00 |  | 10 | 1.49 |  | 28 | 1.15 |  | 9 | **4.51** | ***** |
| **Expanded CVD** | 411 | 1.00 |  | 59 | **2.26** | ***** | 130 | **1.52** | ***** | 31 | **3.46** | ***** |

**Table A4.** All-cause mortality risk for chronic obstructive pulmonary disease (COPD) in the participants with two rounds of follow up. The impact of COPD on all cause-mortality are listed by different COPD stage and gender.

| **Cause of death** |  | **Non-COPD** | | |  | **COPD Stage 1** | | | | |  | **COPD Stage 2** | | | | |  | **COPD Stage 3** | | | | |  | **COPD Stage 4** | | | | |
| --- | --- | --- | --- | --- | --- | --- | --- | --- | --- | --- | --- | --- | --- | --- | --- | --- | --- | --- | --- | --- | --- | --- | --- | --- | --- | --- | --- | --- |
|  |  | n | Death | HR |  | n | Death | HR | 95% CI | |  | n | Death | HR | 95% CI | |  | n | Death | HR | 95% CI | |  | n | Death | HR | 95% CI | |
| **All** |  |  |  |  |  |  |  |  |  |  |  |  |  |  |  |  |  |  |  |  |  |  |  |  |  |  |  |  |
| 1st |  | 155955 | 2,496 | 1.00 |  | 1729 | 156 | **1.15** | (1.02 | , 1.29) |  | 6036 | 344 | **1.40** | (1.29 | , 1.52) |  | 2108 | 154 | **1.69** | (1.51 | , 1.89) |  | 466 | 64 | **2.42** | (2.06 | , 2.85) |
| 2nd |  | 158679 | 2,503 | 1.00 |  | 1460 | 123 | 1.00 | (0.81 | , 1.23) |  | 4862 | 349 | **1.63** | (1.43 | , 1.86) |  | 1639 | 146 | **1.67** | (1.36 | , 2.04) |  | 435 | 62 | **2.35** | (1.76 | , 3.14) |
| **Male** |  |  |  |  |  |  |  |  |  |  |  |  |  |  |  |  |  |  |  |  |  |  |  |  |  |  |  |  |
| 1st |  | 75821 | 1,515 | 1.00 |  | 974 | 108 | 1.12 | (0.97 | , 1.29) |  | 3022 | 238 | **1.45** | (1.32 | , 1.60) |  | 1007 | 112 | **1.75** | (1.53 | , 2.00) |  | 242 | 46 | **2.63** | (2.16 | , 3.21) |
| 2nd |  | 76965 | 1,529 | 1.00 |  | 833 | 78 | 0.89 | (0.68 | , 1.15) |  | 2535 | 250 | **1.67** | (1.44 | , 1.95) |  | 818 | 105 | **1.79** | (1.41 | , 2.28) |  | 229 | 40 | **2.46** | (1.74 | , 3.47) |
| **Female** |  |  |  |  |  |  |  |  |  |  |  |  |  |  |  |  |  |  |  |  |  |  |  |  |  |  |  |  |
| 1st |  | 80134 | 981 | 1.00 |  | 755 | 48 | 1.22 | (1.00 | , 1.50) |  | 3014 | 106 | **1.30** | (1.12 | , 1.50) |  | 1101 | 42 | **1.30** | (1.12 | , 1.50) |  | 224 | 18 | **2.07** | (1.56 | , 2.76) |
| 2nd |  | 81714 | 974 | 1.00 |  | 627 | 45 | 1.31 | (0.92 | , 1.84) |  | 2327 | 99 | **1.57** | (1.23 | , 2.00) |  | 821 | 41 | 1.42 | (0.99 | , 2.05) |  | 206 | 22 | **2.15** | (1.27 | , 3.66) |

**Table A5.** The comparison of mortality risk by amount of leisure time exercise and smoking status between participants with or without COPD.

| **Cause of death** | **Leisure time exercise** | | | | | | | | | | | | | | | | | |
| --- | --- | --- | --- | --- | --- | --- | --- | --- | --- | --- | --- | --- | --- | --- | --- | --- | --- | --- |
|  |  | **Inactive** | | | |  | **Low Active** | | | | |  |  | **Fully Active** | | | | |
|  | n | Death | HR | 95% CI | | n | Death | HR |  | 95% CI | |  | n | Death | HR |  | 95% CI | |
| **All causes** |  |  |  |  |  |  |  |  |  |  |  |  |  |  |  |  |  |  |
| Non-COPD | 175246 | 2,996 | 1.00 | - | | 74665 | 1083 | 0.90 | # | (0.8 | , 1.0) |  | 79279 | 1846 | 0.79 | # | (0.7 | , 0.8) |
| COPD | 9203 | 867 | 1.00 | - | | 4068 | 302 | 0.98 |  | (0.9 | , 1.1) |  | 5003 | 534 | 0.76 | # | (0.7 | , 0.9) |
| **All cancer** |  |  |  |  |  |  |  |  |  |  |  |  |  |  |  |  |  |  |
| Non-COPD | 175246 | 1,305 | 1.00 | - | | 74665 | 493 | 0.94 |  | (0.8 | , 1.0) |  | 79279 | 863 | 0.85 | # | (0.8 | , 0.9) |
| COPD | 9203 | 280 | 1.00 | - | | 4068 | 108 | 1.09 |  | (0.9 | , 1.4) |  | 5003 | 180 | 0.81 | # | (0.7 | , 1.0) |
| **Cardiovascular disease** |  |  |  |  |  |  |  |  |  |  |  |  |  |  |  |  |  |  |
| Non-COPD | 175246 | 506 | 1.00 | - | | 74665 | 175 | 0.83 | # | (0.7 | , 1.0) |  | 79279 | 330 | 0.70 | # | (0.6 | , 0.8) |
| COPD | 9203 | 194 | 1.00 | - | | 4068 | 54 | 0.72 |  | (0.5 | , 1.0) |  | 5003 | 130 | 0.77 | # | (0.6 | , 1.0) |
| **Respiratory system** |  |  |  |  |  |  |  |  |  |  |  |  |  |  |  |  |  |  |
| Non-COPD | 175246 | 110 | 1.00 | - | | 74665 | 30 | 0.69 |  | (0.4 | , 1.1) |  | 79279 | 72 | 0.64 | # | (0.5 | , 0.9) |
| COPD | 9203 | 107 | 1.00 | - | | 4068 | 37 | 1.03 |  | (0.7 | , 1.5) |  | 5003 | 64 | 0.73 |  | (0.5 | , 1.0) |
| **Expanded CVD** |  |  |  |  |  |  |  |  |  |  |  |  |  |  |  |  |  |  |
| Non-COPD | 175246 | 721 | 1.00 | - | | 74665 | 254 | 0.84 | # | (0.7 | , 1.0) |  | 79279 | 452 | 0.69 | # | (0.6 | , 0.8) |
| COPD | 9203 | 284 | 1.00 | - | | 4068 | 82 | 0.80 |  | (0.6 | , 1.0) |  | 5003 | 179 | 0.76 | # | (0.6 | , 0.9) |

**Table A6. COPD and Heart rate according to exercise**

|  | **Total** | |  | **Non-COPD** | |  | **COPD** | |  | **COPD stages** | | | | | | | | | | |
| --- | --- | --- | --- | --- | --- | --- | --- | --- | --- | --- | --- | --- | --- | --- | --- | --- | --- | --- | --- | --- |
|  | **No.** | **%** |  | No. | % |  | No. | % |  | Stage 1 | |  | Stage 2 | |  | Stage 3 | |  | Stage 4 | |
|  |  |  |  |  |  |  |  |  |  | No. | % |  | No. | % |  | No. | % |  | No. | % |
| **Heart Rate (Inactive exercise)** | |  |  |  |  |  |  |  |  |  |  |  |  |  |  |  |  |  |  |  |
| 40-59 | 14,804 | (6.7) |  | 11,825 | (6.8) |  | 577 | (6.3) |  | 91 | (6.4) |  | 340 | (6.6) |  | 110 | (5.5) |  | 36 | (6.2) |
| 60-69 | 62,016 | (28.1) |  | 50,292 | (28.7) |  | 2,215 | (24.1) |  | 416 | (29.1) |  | 1276 | (24.6) |  | 413 | (20.7) |  | 110 | (18.9) |
| 70-79 | 83,823 | (38.0) |  | 67,385 | (38.5) |  | 3,383 | (36.8) |  | 505 | (35.3) |  | 1933 | (37.3) |  | 745 | (37.3) |  | 200 | (34.3) |
| 80-89 | 43,243 | (19.6) |  | 33,453 | (19.1) |  | 2,105 | (22.9) |  | 294 | (20.5) |  | 1194 | (23.1) |  | 476 | (23.8) |  | 141 | (24.2) |
| 90-99 | 12,533 | (5.7) |  | 9,243 | (5.3) |  | 641 | (7.0) |  | 91 | (6.4) |  | 315 | (6.1) |  | 180 | (9.0) |  | 55 | (9.4) |
| 100-109 | 3,209 | (1.5) |  | 2,220 | (1.3) |  | 196 | (2.1) |  | 26 | (1.8) |  | 86 | (1.7) |  | 59 | (3.0) |  | 25 | (4.3) |
| 110-139 | 950 | (0.4) |  | 632 | (0.4) |  | 76 | (0.8) |  | 9 | (0.6) |  | 34 | (0.7) |  | 17 | (0.9) |  | 16 | (2.7) |
| 80-99 | 55,776 | (25.3) |  | 42,696 | (24.4) |  | 2,746 | (29.9) |  | 385 | (26.9) |  | 1,509 | (29.1) |  | 656 | (32.8) |  | 196 | (33.6) |
| **Heart Rate (Active exercise)** | |  |  |  |  |  |  |  |  |  |  |  |  |  |  |  |  |  |  |  |
| 40-59 | 18,480 | (9.6) |  | 15,340 | (10.0) |  | 778 | (8.6) |  | 189 | (10.5) |  | 447 | (8.9) |  | 115 | (6.6) |  | 27 | (5.7) |
| 60-69 | 60,324 | (31.4) |  | 49,667 | (32.3) |  | 2,463 | (27.2) |  | 537 | (29.8) |  | 1374 | (27.2) |  | 434 | (24.9) |  | 118 | (25.1) |
| 70-79 | 68,624 | (35.8) |  | 55,022 | (35.8) |  | 3,238 | (35.7) |  | 607 | (33.7) |  | 1851 | (36.7) |  | 625 | (35.8) |  | 155 | (32.9) |
| 80-89 | 32,433 | (16.9) |  | 25,022 | (16.3) |  | 1,841 | (20.3) |  | 355 | (19.7) |  | 994 | (19.7) |  | 387 | (22.2) |  | 105 | (22.3) |
| 90-99 | 9,109 | (4.7) |  | 6,669 | (4.3) |  | 554 | (6.1) |  | 87 | (4.8) |  | 291 | (5.8) |  | 136 | (7.8) |  | 40 | (8.5) |
| 100-109 | 2,268 | (1.2) |  | 1,636 | (1.1) |  | 144 | (1.6) |  | 14 | (0.8) |  | 73 | (1.4) |  | 36 | (2.1) |  | 21 | (4.5) |
| 110-139 | 637 | (0.3) |  | 448 | (0.3) |  | 45 | (0.5) |  | 11 | (0.6) |  | 18 | (0.4) |  | 11 | (0.6) |  | 5 | (1.1) |
| 80-99 | 41,542 | (21.7) |  | 31,691 | (20.6) |  | 2,395 | (26.4) |  | 442 | (24.6) |  | 1,285 | (25.5) |  | 523 | (30.0) |  | 145 | (30.8) |

| **Table A7.** Characteristics of participants and cause-specific mortality by heart rate for study cohort | | | | | | | | | | | | | | | | | | | | | | | | | | | | |
| --- | --- | --- | --- | --- | --- | --- | --- | --- | --- | --- | --- | --- | --- | --- | --- | --- | --- | --- | --- | --- | --- | --- | --- | --- | --- | --- | --- | --- |
|  |  | 40-59 | | | | 60-69 | | 70-79 | | | | 80-89 | | | | 90-99 | | | | ≥ 100 | | | | 80-99 | | | | Total |
|  |  | N | | (%) | | N | (%) | N | | (%) | | N | | (%) | | N | | (%) | | N | | (%) | | N | | (%) | |  |
| All participants |  | 42,910 | | (8.1) | | 158,060 | (29.7) | 196,498 | | (36.9) | | 97,877 | | (18.4) | | 28,189 | | (5.3) | | 9,374 | | (1.8) | | 126,066 | | (23.7) | | 532,908 |
|  |  |  |  |  |  |  |  |  |  |  |  |  |  |  |  |  |  |  |  |  |  |  |  |  |  |  |  |  |
| Gender | Male | 27,646 | | (10.9) | | 82,740 | (32.6) | 86,824 | | (34.2) | | 40,870 | | (16.1) | | 11,771 | | (4.6) | | 3,795 | | (1.5) | | 52,641 | | (20.8) | | 253,646 |
|  | Female | 15,264 | | (5.5) | | 75,320 | (27.0) | 109,674 | | (39.3) | | 57,007 | | (20.4) | | 16,418 | | (5.9) | | 5,579 | | (2.0) | | 73,425 | | (26.3) | | 279,262 |
| Age (years) | 20-39 | 22,750 | | (7.9) | | 85,254 | (29.7) | 107,133 | | (37.4) | | 52,650 | | (18.4) | | 14,457 | | (5.0) | | 4,470 | | (1.6) | | 67,107 | | (23.4) | | 286,714 |
|  | 40-59 | 13,268 | | (7.6) | | 52,154 | (29.7) | 65,470 | | (37.3) | | 32,182 | | (18.4) | | 9,181 | | (5.2) | | 3,100 | | (1.8) | | 41,363 | | (23.6) | | 175,355 |
|  | ≥ 60 | 6,892 | | (9.7) | | 20,652 | (29.2) | 23,895 | | (33.7) | | 13,045 | | (18.4) | | 4,551 | | (6.4) | | 1,804 | | (2.5) | | 17,596 | | (24.8) | | 70,839 |
|  |  | Deaths | HR | (95%CI) | | Deaths | HR | Deaths | HR | (95%CI) | | Deaths | HR | (95%CI) | | Deaths | HR | (95%CI) | | Deaths | HR | (95%CI) | | Deaths | HR | (95%CI) | | Deaths |
| All-cause mortality |  | 1,229 | 0.98 | (0.91 | ,1.06) | 4319 | 1.00 | 6,189 | 1.09 | (1.03 | ,1.14) | 4,200 | 1.30 | (1.23 | ,1.37) | 1,794 | 1.74 | (1.62 | ,1.88) | 941 | 2.45 | (2.23 | ,2.69) | 5,994 | 1.40 | (1.33 | ,1.48) | 18,672 |
| Cardiovascular disease (CVD) |  | 270 | 0.93 | (0.78 | ,1.12) | 877 | 1.00 | 1,203 | 1.04 | (0.93 | ,1.17) | 867 | 1.32 | (1.17 | ,1.50) | 351 | 1.60 | (1.36 | ,1.89) | 189 | 2.08 | (1.66 | ,2.59) | 1,218 | 1.40 | (1.25 | ,1.58) | 3,757 |
| Stroke |  | 99 | 0.73 | (0.54 | ,0.99) | 400 | 1.00 | 533 | 1.03 | (0.86 | ,1.22) | 370 | 1.20 | (0.99 | ,1.46) | 145 | 1.48 | (1.14 | ,1.92) | 76 | 1.81 | (1.26 | ,2.58) | 515 | 1.27 | (1.06 | ,1.52) | 1,623 |
| Ischemic heart disease |  | 85 | 1.35 | (0.98 | ,1.85) | 208 | 1.00 | 327 | 1.23 | (0.98 | ,1.53) | 227 | 1.49 | (1.17 | ,1.89) | 99 | 1.61 | (1.16 | ,2.23) | 51 | 2.61 | (1.76 | ,3.87) | 326 | 1.55 | (1.23 | ,1.94) | 997 |
| Expanded CVD |  | 344 | 0.95 | (0.81 | ,1.11) | 1160 | 1.00 | 1,720 | 1.11 | (1.01 | ,1.23) | 1,363 | 1.43 | (1.29 | ,1.59) | 626 | 1.87 | (1.64 | ,2.14) | 323 | 2.30 | (1.94 | ,2.73) | 1,989 | 1.55 | (1.41 | ,1.71) | 5,536 |
| Diabetes mellitus |  | 41 | 0.80 | (0.51 | ,1.28) | 191 | 1.00 | 357 | 1.37 | (1.08 | ,1.75) | 362 | 1.90 | (1.48 | ,2.44) | 200 | 2.66 | (2.01 | ,3.52) | 94 | 2.74 | (1.93 | ,3.89) | 562 | 2.14 | (1.69 | ,2.72) | 1,245 |
| Kidney diseases |  | 33 | 1.30 | (0.74 | ,2.29) | 92 | 1.00 | 160 | 1.44 | (1.00 | ,2.07) | 134 | 1.89 | (1.29 | ,2.78) | 75 | 3.23 | (2.09 | ,4.99) | 40 | 4.89 | (2.96 | ,8.09) | 209 | 2.21 | (1.54 | ,3.17) | 534 |
| Cancer |  | 494 | 0.97 | (0.86 | ,1.09) | 1835 | 1.00 | 2,459 | 1.01 | (0.93 | ,1.09) | 1,498 | 1.18 | (1.08 | ,1.28) | 543 | 1.42 | (1.26 | ,1.61) | 289 | 2.13 | (1.82 | ,2.49) | 2,041 | 1.23 | (1.14 | ,1.34) | 7,118 |
| Liver cancer |  | 139 | 1.28 | (1.01 | ,1.62) | 398 | 1.00 | 506 | 1.00 | (0.85 | ,1.19) | 332 | 1.29 | (1.07 | ,1.55) | 100 | 1.45 | (1.11 | ,1.89) | 63 | 2.29 | (1.64 | ,3.19) | 432 | 1.34 | (1.13 | ,1.60) | 1,538 |
| Lung cancer |  | 115 | 0.96 | (0.75 | ,1.23) | 398 | 1.00 | 533 | 0.97 | (0.83 | ,1.14) | 281 | 1.03 | (0.85 | ,1.24) | 113 | 1.32 | (1.02 | ,1.73) | 60 | 1.72 | (1.19 | ,2.48) | 394 | 1.10 | (0.92 | ,1.31) | 1,500 |
| Digestive system |  | 84 | 1.17 | (0.85 | ,1.61) | 264 | 1.00 | 413 | 1.43 | (1.17 | ,1.76) | 294 | 1.66 | (1.33 | ,2.08) | 131 | 2.22 | (1.66 | ,2.96) | 77 | 3.65 | (2.59 | ,5.14) | 425 | 1.85 | (1.50 | ,2.29) | 1,263 |
| Cirrhosis |  | 43 | 1.05 | (0.68 | ,1.62) | 148 | 1.00 | 209 | 1.27 | (0.97 | ,1.66) | 174 | 1.27 | (0.97 | ,1.66) | 59 | 1.83 | (1.22 | ,2.75) | 37 | 2.96 | (1.81 | ,4.85) | 233 | 1.85 | (1.40 | ,2.44) | 670 |
| Liver disease  (liver cancer or cirrhosis) | | 182 | 1.23 | (1.00 | ,1.51) | 546 | 1.00 | 715 | 1.07 | (0.93 | ,1.24) | 506 | 1.41 | (1.21 | ,1.65) | 159 | 1.55 | (1.24 | ,1.94) | 100 | 2.47 | (1.87 | ,3.25) | 665 | 1.47 | (1.27 | ,1.71) | 2,208 |
| Respiratory system |  | 63 | 0.75 | (0.51 | ,1.08) | 231 | 1.00 | 380 | 1.30 | (1.04 | ,1.62) | 281 | 1.66 | (1.31 | ,2.10) | 168 | 3.11 | (2.36 | ,4.10) | 90 | 4.30 | (3.04 | ,6.09) | 449 | 1.95 | (1.56 | ,2.42) | 1,213 |
| COPD |  | 19 | 0.55 | (0.27 | ,1.12) | 84 | 1.00 | 143 | 1.17 | (0.80 | ,1.72) | 124 | 2.33 | (1.59 | ,3.41) | 84 | 5.55 | (3.68 | ,8.36) | 56 | 7.85 | (4.77 | ,12.93) | 208 | 3.08 | (2.17 | ,4.36) | 510 |
| Suicide |  | 36 | 1.36 | (0.86 | ,2.16) | 102 | 1.00 | 168 | 1.18 | (0.87 | ,1.59) | 93 | 1.33 | (0.94 | ,1.87) | 39 | 1.99 | (1.27 | ,3.14) | 13 | 2.44 | (1.25 | ,4.76) | 132 | 1.47 | (1.07 | ,2.02) | 451 |
| Hazard ratios were adjusted for age, gender, education, BMI, smoking status, physical activity, anemia, blood pressure and blood glucose. | | | | | | | | | | | | | | | | | | | | | | | | | | | | |

**Figure A1.** Flow chart of participants in MJ cohort study.
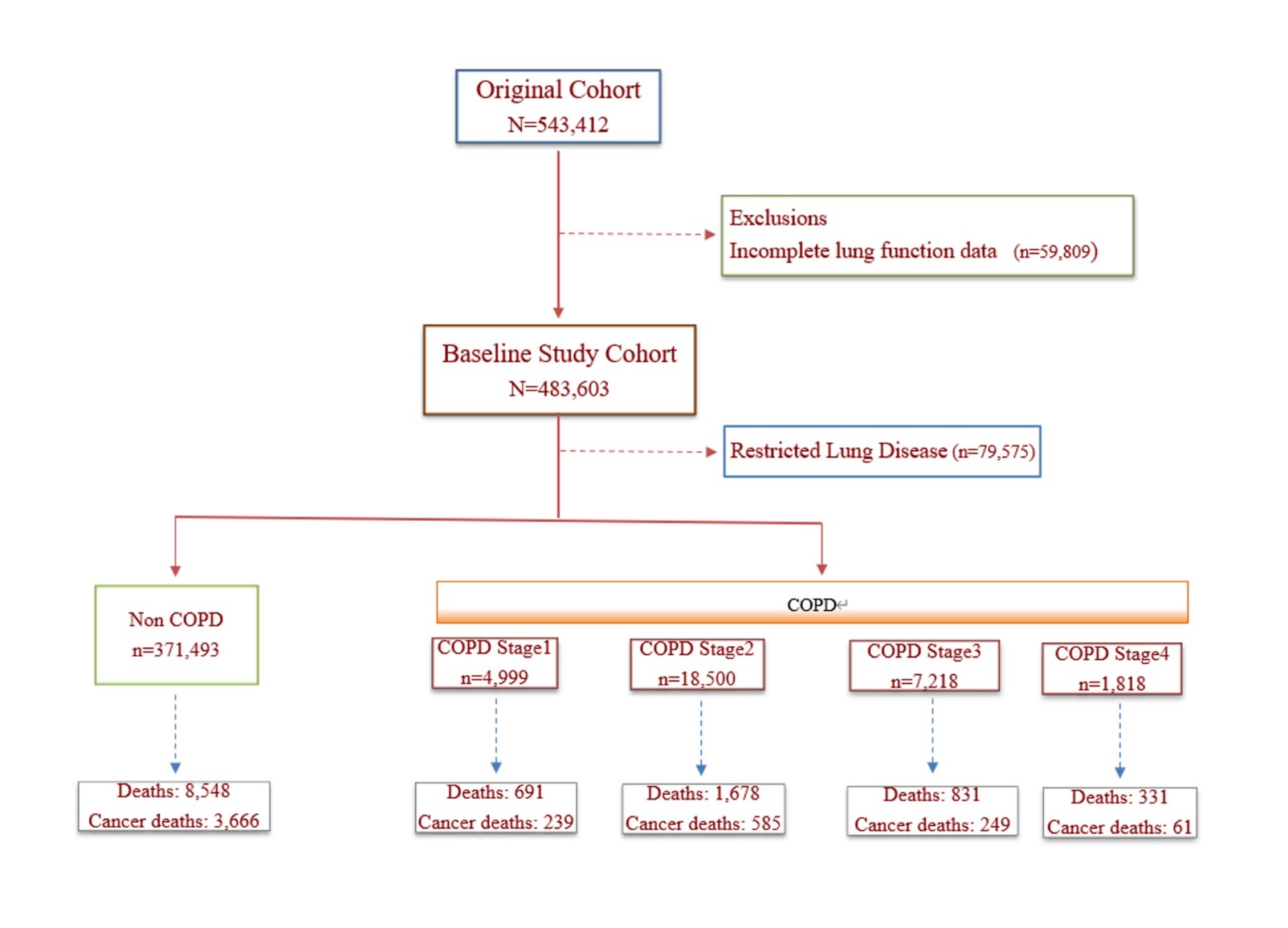


**Figure A2.** Estimated COPD prevalence in Taiwan by gender and smoking status

**
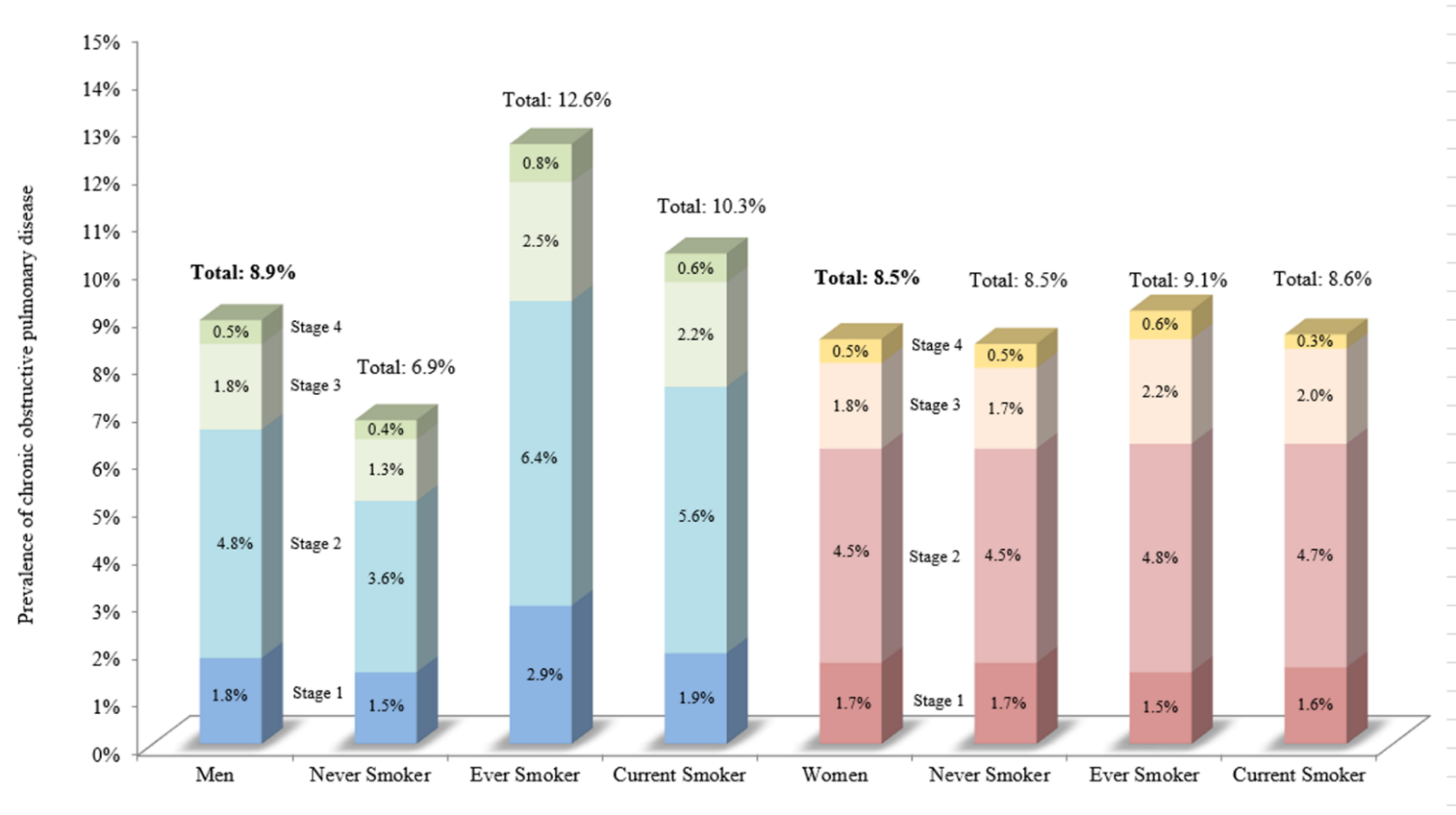
**
